# Staged acute mesenteric and peripheral ischemia treatment in COVID-19 patient: Case report

**DOI:** 10.1016/j.ijscr.2021.106105

**Published:** 2021-06-09

**Authors:** E. Dinoto, F. Ferlito, M.A. La Marca, D. Mirabella, G. Bajardi, F. Pecoraro

**Affiliations:** a Vascular Surgery Unit - AOUP Policlinico ‘P. Giaccone’, Palermo, Italy; b Department of Surgical, Oncological and Oral Sciences – University of Palermo, Italy

**Keywords:** COVID 19, Acute mesenteric ischemia, Acute limb ischemia, Mechanical thrombectomy, Case report

## Abstract

**Introduction:**

COVID-19 is an infectious disease that has been associated not only with respiratory complications. The COVID-19 disease includes, also damage to other organ systems as well as coagulopathy. The present report describes a case of COVID-19 presenting with acute mesenteric ischemia (AMI) and subsequent acute limb ischemia (ALI).

**Presentation of case:**

An 84-years old hospitalized female patient presenting diabetes and recent COVID-19 reported acute onset of abdominal pain and typical findings of AMI. The CT-angiography confirmed the AMI secondary to a superior mesenteric artery (SMA) occlusion. The patient was managed through an endovascular approach using a SMA mechanical thrombectomy and stenting with a good result.

**Discussion:**

Treatment of this life-threatening condition includes surgical resection of the necrotic bowel, restoration of blood flow to the ischemic intestine and supportive measure - gastrointestinal decompression, fluid resuscitation, hemodynamic support. Endovascular management of AMI is preferred over the standard surgical approach due to a reduced mortality and morbidity rates. Imaging findings of intestinal necrosis, however, represent an indication for AMI surgical treatment with explorative laparotomy. Different endovascular solutions have been employed to address AMI including mechanical thrombectomy, local thrombolysis, and PTA-stenting.

**Conclusion:**

COVID-19 clinical presentation can be atypical, including gastrointestinal symptoms. If a first embolic event occurs, an aggressive anticoagulation treatment could be inefficient to reduce the risk of subsequent embolization events. The limited life expectancy of such revascularization procedures should orientate towards less invasive treatments.

## Highlights

• The COVID-19 disease includes damage to other organ systems as well as coagulopathy.
• COVID-19 clinical presentation can be atypical, including gastrointestinal symptoms.
• Endovascular approach of AMI is preferred due to a reduced mortality.
• Few cases of COVID-related AMI have been reported.

## 1 Introduction

Coronavirus disease 19 (COVID-19) pandemic is worldwide affecting more than 150 million people including more than 3 million deaths [1]. COVID-19 has been increasingly associated with thromboembolic complications in both arterial and venous districts. Hyperinflammation, platelet activation, endothelial dysfunction, and stasis have been advocated as predisponent to thrombotic complications [2]. Acute ischemic manifestations in COVID-19 patients are frequently reported, especially in the peripheral district [3]. A single patient presenting consecutive acute mesenteric ischemia (AMI) and subsequent acute limb ischemia (ALI) is reported.

This work has been written in accordance with the SCARE criteria [4].

## 2 Case report

An 84-year-old female with hypertension, diabetes mellitus, severe renal failure (Glomerular filtration rate - GFR - 44 ml/min) was referred to our hospital for fever and dyspnea. Medical history reported gastric ulcer disease with hematemesis and angioplasty for diabetic peripheral artery disease in pharmacological treatment with regular anti-platelet therapy (Cardioaspirin - Bayer, 100 mg/day). No coronary artery disease or cardiac arrhythmias were reported.

At admission, her physical examination revealed a sinusal heart rate of 90 bpm, blood pressure of 105/70 mmHg, fever (37.5 °C), respiratory rate of 20 breaths/min, and oxygen saturation of 89%, abdomen's soft and non-tender in the absence of pain.

**Fig. 1:**
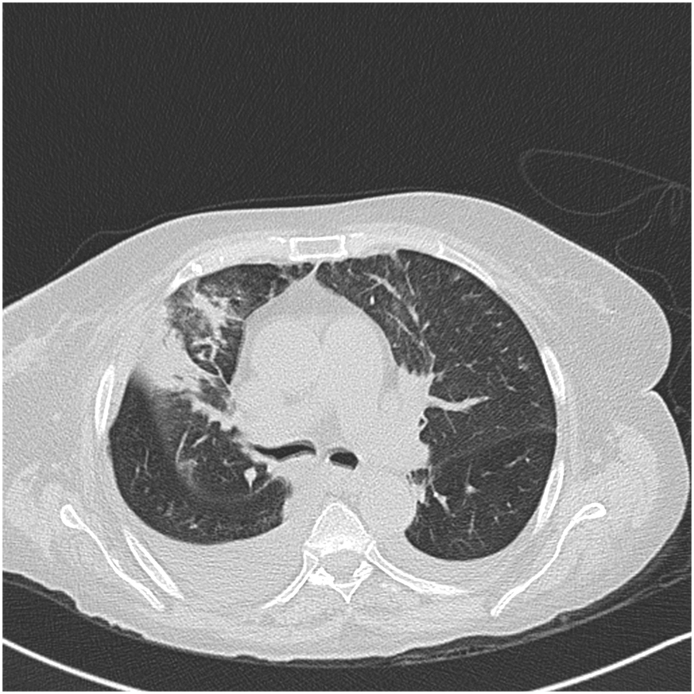
CT-scan showing bilateral distribution of ground glass opacities with consolidation areas in the right lobe.

The pulmonary CT scan showed the typical finding of COVID 19 infection and severe acute respiratory syndrome coronavirus 2 (SARS-CoV-2) with the typical interstitial pneumonia infection (Fig. 1). The rhino-laryngeal swab confirmed the COVID-19 infection, and the patient was transferred to the COVID ward. At 48 h from admission, the patient reported acute onset of abdominal pain and signs of acute abdomen and AMI. At physical examination, abdomen became tense and distened without peristalsis and empty rectum on digital exam. Laboratory data were as follows: increase of aspartate aminotransferase (AST) 38 U/l (*n* = 0–37), alanine aminotransferase (ALT) 216 U/l (*n* = 0–41), lactate dehydrogenase (LDH) - 431 U/l (*n* = 50–250) levels with D-dimer 6937 ng/ml (n = 0–700), C-reactive protein (CPR) 32.47 mg/dl (*n* ≤ 5) and White blood cell (WBC) 18.000/μl (neutrophils: 80.5%) (*n* = 4–11 neutrophils: 40–74%). Renal function impairment was evident with a reported GFR of 30 ml/min (*n* ≥ 90).

**Fig. 2:**
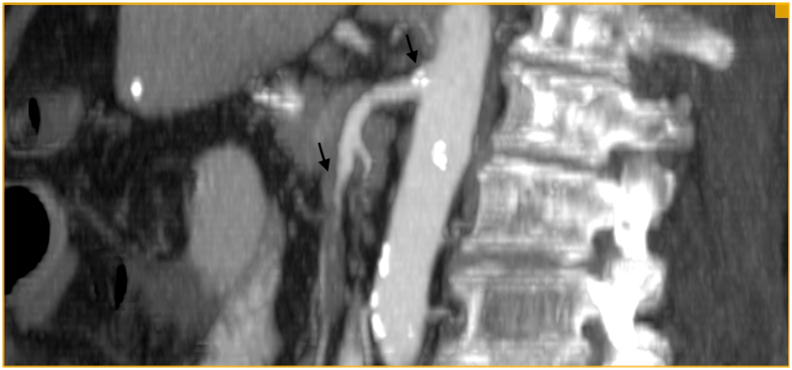
Preoperative CT-angiography showing superior mesenteric artery (SMA) origin stenosis and occlusion after 2 cm from the origin.

**Fig. 3:**
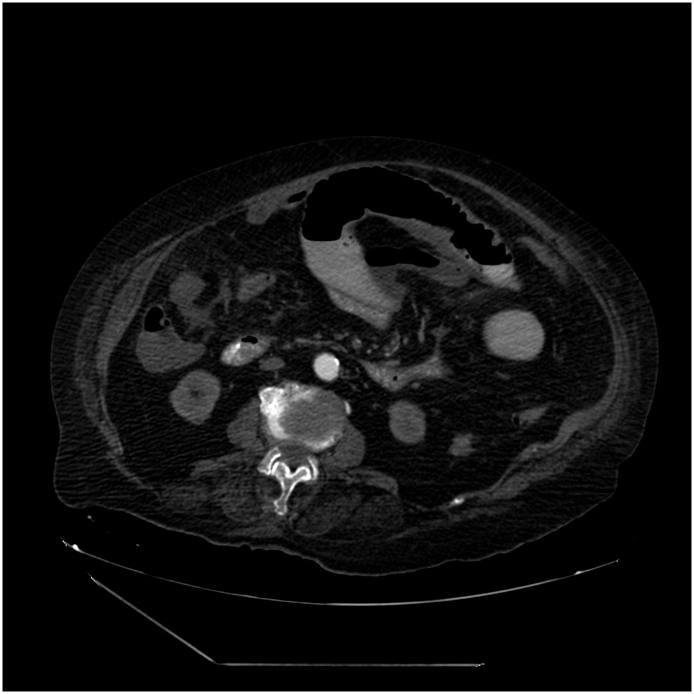
CT-scan showing Early stage of Mesenteric Ischemia with absence of bowel mural enhancement.

Abdominal CT-angiography showed superior mesenteric artery (SMA) origin stenosis and occlusion after 2 cm from the origin (Fig. 2); CT findings of AMI at an early stage were reported including the absence of bowel mural enhancement in the proximal part of the ileum. No intestinal necrosis findings were reported (Fig. 3).

**Fig. 4:**
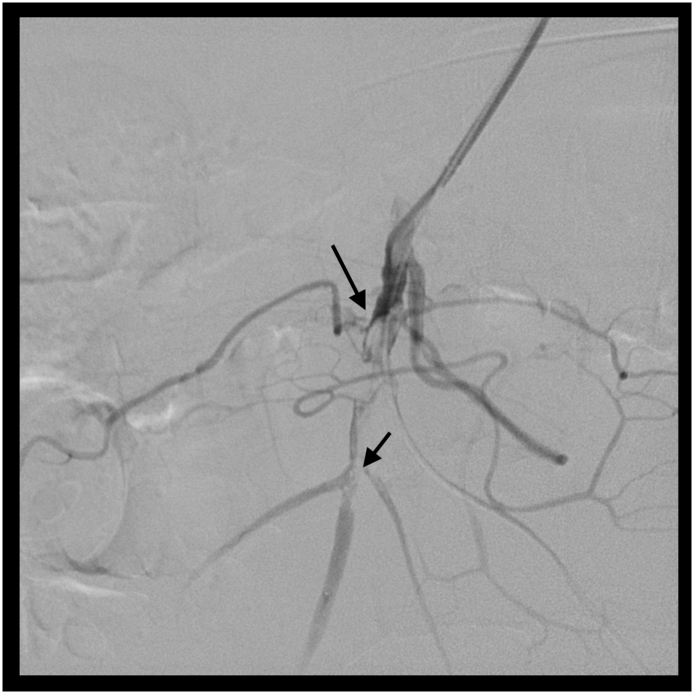
Diagnostic Intraoperative angiography showing thrombus in superior mesenteric artery.

A surgical attempt with SMA thrombectomy was excluded due to the high surgical risk mainly related to respiratory impairment and on this basis, an endovascular approach was chosen (Fig. 4).

**Fig. 5:**
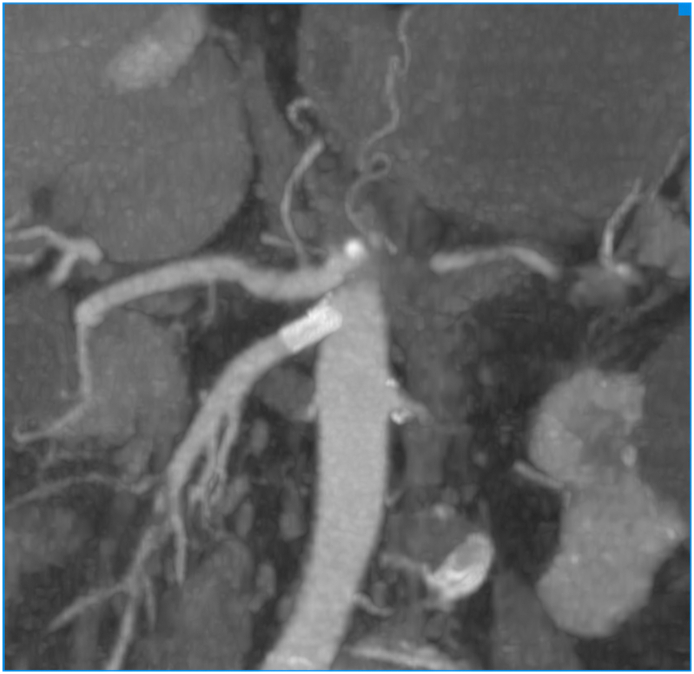
Post-operative CT-angiography showing superior mesenteric artery (SMA) after proximal stunting and mechanical thrombectomy.

The treatment consisted of percutaneous trans-brachial access simultaneous mechanical thrombectomy using a 6F catheter Export AP Aspiration Catheter (Medtronic Minneapolis, MN) and proximal SMA balloon-expandable uncovered stenting (6 × 20 mm Isthmus, CID SpA, IT) with SMA patency restoration (Fig. 5).

In brief, the SMA was cannulated using a reverse-curve catheter and a hydrophilic 0·035-inch guidewire, which is placed into the ileocolic branch of the SMA. The wire is then replaced with a stiffer, Amplatz (Boston Scientific - Natick, Massachusetts) or Rosen (Cook Inc. - Bloomington, IN) to achieve stability and a 6-Fr/45 cm Destination (Terumo Corporation, Tokyo, Japan) introducer placed proximal to the embolus in the SMA. Inside the long introducer, a 6-Fr guiding catheter is positioned into the clot, which is aspirated with a 20-ml syringe while the introducer is withdrawn. In presence of thrombus, usually, stenting is contraindicated but it can be used in presence of calcification or to preserve primary patency.

At 24 h from the index operation, the CT-angiography confirmed SMA patency with a non-significant residual thrombus on the artery wall and maintenance of SMA distal flow.

Clinical and laboratory findings were suggestive for AMI regression due to abdominal pain resolution, aspartate aminotransferase (AST) 20 U/l (*n* = 0–37), alanine aminotransferase (ALT) 45 U/l (n = 0–41), lactate dehydrogenase (LDH) - 355 U/l (*n* = 50–250), levels with D-dimer 2976 ng/ml (n = 0–700), C-reactive protein (CPR) 35.31 mg/dl (*n* ≤5) and White blood cell (WBC) 15.000/μl (neutrophils: 71.5%) (*n* = 4–11 neutrophils: 40–74%).

Postoperative medical management consisted of dual antiplatelet therapy (Cardioaspirin 100 mg plus Clopidogrel 75 mg) and low molecular weight heparin (LMWH) from day one. Echocardiogram was negative for cardiac thrombus.

On the 7th postoperative day, the patient presented right leg rest pain and foot cyanosis. These typical findings of acute limb ischemia (ALI) were confirmed on duplex ultrasound (DUS) showing a right femoropopliteal artery thrombosis.

The right ALI was managed under local anesthesia by hybrid surgical transfemoral thrombectomy and distal superficial femoral artery stenting due to a residual dissection.

At 24 h from the second procedure the leg appeared normothermic with a good popliteal pulse, the DUS confirmed patent femoropopliteal arteries. At the same time, the patient showed a significant decline of respiratory function requiring intensive care management and death on the 5th postoperative day from the second intervention due to respiratory acute failure.

## 3 Discussion

The usual clinical presentation of COVID-19 is fever and respiratory symptoms. Abdominal symptoms are also a common finding in COVID-19 patients and have been reported in 16% of cases [5]. Less frequently COVID-19 patients present with arterial or venous occlusion in different districts including AMI and ALI [6]. Vaccination modeling for safe surgeries and identification of timing of surgery in COVID-19 patients are currently under investigation [7,8].

Treatment of this life-threatening condition includes surgical resection of the necrotic bowel, restoration of blood flow to the ischemic intestine and supportive measure - gastrointestinal decompression, fluid resuscitation, hemodynamic support [9]. Endovascular management of AMI is preferred over the standard surgical approach due to a reduced mortality and morbidity rates [10]. Imaging findings of intestinal necrosis, however, represent an indication for AMI surgical treatment with explorative laparotomy [11]. Different endovascular solutions have been employed to address AMI including mechanical thrombectomy, local thrombolysis, and PTA-stenting. In the reported patient, local thrombolysis was excluded due to the renal function impairment and the related risk of bleeding. The lack of peritonitis allowed for mechanical thrombectomy [12,13]. In addition, a proximal SMA angioplasty with a stent was chosen to increase blood flow and achieve full revascularization. Acute-on-chronic peripheral arterial disease in patients presenting diabetes mellitus is frequent also in the general population [14]. The successful hybrid treatment with standard embolectomy and distal SFA stenting was employed to reduce operative invasiveness [15].

Few cases of COVID-related AMI have been reported [16], but its association with ALI is new.

An elevated D-dimer level has been identified as a thromboembolism marker and it has associated with unfavorable prognosis [17], also in COVID-19 patients and as reported in our case.

## 4 Conclusions

The consecutive AMI and ALI presentation is a new finding in COVID-19 patients leading to a careful evaluation in those patients with the potential of presenting consecutive embolic events. In circumstances when a first embolic event occurs, D-Dimer evaluation and aggressive anticoagulation treatment should be advocated. The associated poor outcomes of AMI treatment indicate less invasive endovascular management especially in COVID-19 patients presenting a reduced life expectancy.

## Funding

None.

## Ethical approval

Not applicable.

## Consent

Written informed consent was obtained from the patient for publication of this case report and accompanying images.

## Author contribution

Ettore Dinoto: study concept, design, data collection, data analysis, interpretation, writing the paper, final approval of the version to be submitted, guarantor.

Felice Pecoraro: study concept, design, data collection, data analysis, interpretation, writing the paper, final approval of the version to be submitted.

Francesca Ferlito: study concept, design, data collection, data analysis, interpretation, final approval of the version to be submitted.

Manfredi Agostino La Marca: study concept, design, data collection, final approval of the version to be submitted.

Domenico Mirabella: study concept, design, data collection, final approval of the version to be submitted.

Guido Bajardi: study concept, design, data collection, data analysis, interpretation, final approval of the version to be submitted.

## Guarantor

Ettore Dinoto.

## International journal of surgery case reports

The following information is required for submission. Please note that failure to respond to these questions/statements will mean your submission will be returned. If you have nothing to declare in any of these categories, then this should be stated.

## Registration of research studies

Not applicable.

## Declaration of competing interest

The authors have no ethical conflicts to disclose.
